# High-throughput identification of novel conotoxins from the Chinese tubular cone snail (*Conus betulinus*) by multi-transcriptome sequencing

**DOI:** 10.1186/s13742-016-0122-9

**Published:** 2016-04-14

**Authors:** Chao Peng, Ge Yao, Bing-Miao Gao, Chong-Xu Fan, Chao Bian, Jintu Wang, Ying Cao, Bo Wen, Yabing Zhu, Zhiqiang Ruan, Xiaofei Zhao, Xinxin You, Jie Bai, Jia Li, Zhilong Lin, Shijie Zou, Xinhui Zhang, Ying Qiu, Jieming Chen, Steven L. Coon, Jiaan Yang, Ji-Sheng Chen, Qiong Shi

**Affiliations:** BGI-Shenzhen, Shenzhen, 518083 China; Research Institute of Pharmaceutical Chemistry, Beijing, 102205 China; School of Pharmaceutical Sciences, Hainan Medical University, Haikou, 571199 China; Shenzhen Key Laboratory of Marine Genomics, Guangdong Provincial Key Laboratory of Molecular Breeding in Marine Economic Animals, State Key Laboratory of Agricultural Genomics, Shenzhen, 518083 China; Micro Pharmatech Ltd, Wuhan, 430075 China; Molecular Genomics Laboratory, National Institutes of Health, Bethesda, MD 20892 USA; BGI-Zhenjiang Institute of Hydrobiology, Zhenjiang, 212000 China

**Keywords:** Cone snail, Conopeptide, Conotoxin, *Conus betulinus*, Transcriptome, Venom duct, Venom bulb

## Abstract

**Background:**

The venom of predatory marine cone snails mainly contains a diverse array of unique bioactive peptides commonly referred to as conopeptides or conotoxins. These peptides have proven to be valuable pharmacological probes and potential drugs because of their high specificity and affinity to important ion channels, receptors and transporters of the nervous system. Most previous studies have focused specifically on the conopeptides from piscivorous and molluscivorous cone snails, but little attention has been devoted to the dominant vermivorous species.

**Results:**

The vermivorous Chinese tubular cone snail, *Conus betulinus*, is the dominant *Conus* species inhabiting the South China Sea. The transcriptomes of venom ducts and venom bulbs from a variety of specimens of this species were sequenced using both next-generation sequencing and traditional Sanger sequencing technologies, resulting in the identification of a total of 215 distinct conopeptides. Among these, 183 were novel conopeptides, including nine new superfamilies. It appeared that most of the identified conopeptides were synthesized in the venom duct, while a handful of conopeptides were identified only in the venom bulb and at very low levels.

**Conclusions:**

We identified 215 unique putative conopeptide transcripts from the combination of five transcriptomes and one EST sequencing dataset. Variation in conopeptides from different specimens of *C. betulinus* was observed, which suggested the presence of intraspecific variability in toxin production at the genetic level. These novel conopeptides provide a potentially fertile resource for the development of new pharmaceuticals, and a pathway for the discovery of new conotoxins.

**Electronic supplementary material:**

The online version of this article (doi:10.1186/s13742-016-0122-9) contains supplementary material, which is available to authorized users.

## Background

The genus *Conus* is classified in the Coninae subfamily within the Conidae family that belongs to the Conoidea superfamily (a branch of the Neogastropoda clade) [1, 2]. With an estimation of 700 species [3, 4], all cone snails are classified in *Conus*, which is the largest genus among marine invertebrates. Venomous cone snails are carnivorous and predatory marine gastropod mollusks that use a complex cocktail of venom components for many reasons, including capture and digestion of prey, defense against foes [5], avoidance of competitors and other biological purposes [6]. According to variation in diet, cone snails are divided into three groups: piscivorous species that hunt small fish, molluscivorous species that feed on other marine snails including other cone snails, and vermivorous cone snails that prey on polychaetes and hemichordates [7].

As slow-moving predatory marine gastropods, cone snails have developed successful strategies to subdue quicker or stronger prey during more than 55 million years of evolution [8], including a hollow, harpoon-like radular tooth and potent toxins targeted to the nervous system and musculature of the prey [9]. The modified radular tooth (along with a venom gland) can be launched out from the snail’s mouth deep into the prey’s flesh in a harpoon-like action. The injected venom rapidly enters the victim’s circulatory system, interacts with a range of molecular targets in the nervous system, and causes paralysis in a short time (sometimes within a few seconds) [10, 11].

In contrast to the well-known large protein toxins in snake venom, *Conus* venom mainly contains a diverse array of unique bioactive peptides commonly referred to as conopeptides or conotoxins. These small polypeptide conotoxins typically range from seven to 46 amino acids in length, with many of them consisting of 12–30 amino acids [12]. They have high specificity and affinity to voltage-gated ion channels, ligand-gated ion channels, G-protein-coupled receptors and neurotransmitter transporters in the central and peripheral nervous systems [3, 6, 12–18]. Because of their bioactive specificity, *Conus* venoms have become a potent resource for pharmacological neuroscience research [19–22] and a promising source for the discovery of new drugs to treat a wide variety of human neurological diseases [6, 23–30]. To date, several conotoxins have already demonstrated potential therapeutic effects in preclinical or clinical trials. The most well-known is ω-MVIIA (commercially known as ziconotide), derived from the venom of *C. magus*, which has been approved by the US Food and Drug Administration (FDA) to treat previously unmanageable chronic pain in cancer and AIDS patients [30–32]. Another conotoxin, conantokin G, a specific antagonist against the NR2B subunit of the NMDA receptor, is in human clinical trials for intractable epilepsy [33]. In addition, more and more conopeptides are undergoing development for the treatment of pathologies including pain, Parkinson’s disease, cardiac infarction, hypertension and various neurological diseases [12, 28, 34–37].

With a few exceptions, each conopeptide precursor generally consists of three distinct regions: a highly conserved N-terminal signal peptide region, a less conserved intervening propeptide region, and a hypervariable C-terminal mature toxin region [38, 39]. Based on the sequence similarities of signal peptides in the precursors [40], conopeptides are currently classified into 26 gene superfamilies (A, B1, B2, B3, C, D, E, F, G, H, I1, I2, I3, J, K, L, M, N, O1, O2, O3, P, S, T, V and Y) [41–47] and 13 temporary gene superfamilies for those identified in the early divergent clade species [40, 42, 48, 49]. Although amino acid conservation in the mature peptide sequences of conopeptides within a same gene superfamily is much lower than in the signal and propeptide regions, certain characteristic cysteine frameworks within the mature conotoxins are often (but not always) specific to a conotoxin superfamily. So far, 26 distinct cysteine frameworks have been described, and they may be associated with particular pharmacological families [39, 40, 50].

Early views of the conotoxin-producing structures concluded that the muscular bulbous organ (the venom bulb, located at the end of the venom duct) was likely to participate in venom biosynthesis [51]. With the development of molecular biology, researchers found that the epithelial cells lining the cone snail’s venom duct were rich in mature mRNAs encoding precursor conopeptides, and the venom bulb may function to propel the venom toward the pharynx while preying or defending [51–53].

With early estimates of an average of 100 conotoxins per species, recent reports proved the presence of 1000 to 2000 different conopeptides in a single sample of venom using high-sensitivity mass spectrometry [44, 54, 55]. A single mutation in a mature sequence can add one more conopeptide at the DNA level and, subsequently, owing to the 14 different post-translational modifications known in addition to other undefined alterations, an average of 20 different toxin variants for each conopeptide precursor can be characterized at the protein level [44]. This raises the possibility that the total of 500 to 700 species of cone snails, providing upwards of 50,000 conopeptide genes and 1,000,000 mature conotoxins as potential pharmacological targets, constitutes the largest single library of natural drug candidates.

Despite the large number of potential conopeptide genes and mature conotoxins, only approximately 1400 nucleotide sequences of conotoxin genes have been reported from 100 *Conus* species by traditional approaches over the past decades, with as few as 210 peptides being validated at the protein level [40, 41]. Traditional methods, which may isolate and sequence these potential bioactives, are generally time-consuming, of low sensitivity, and often limited by sample availability. In contrast, high-throughput sequencing can achieve greater sequencing depth and larger coverage of the transcriptome so that even rare transcripts with low expression levels can be identified [56]. Recent studies on the venom duct transcriptome of several *Conus* species, using next-generation sequencing technologies, have uncovered about 100 conopeptide genes per *Conus* species [5, 44, 53, 57–62].

## Data description

**Fig. 1:**
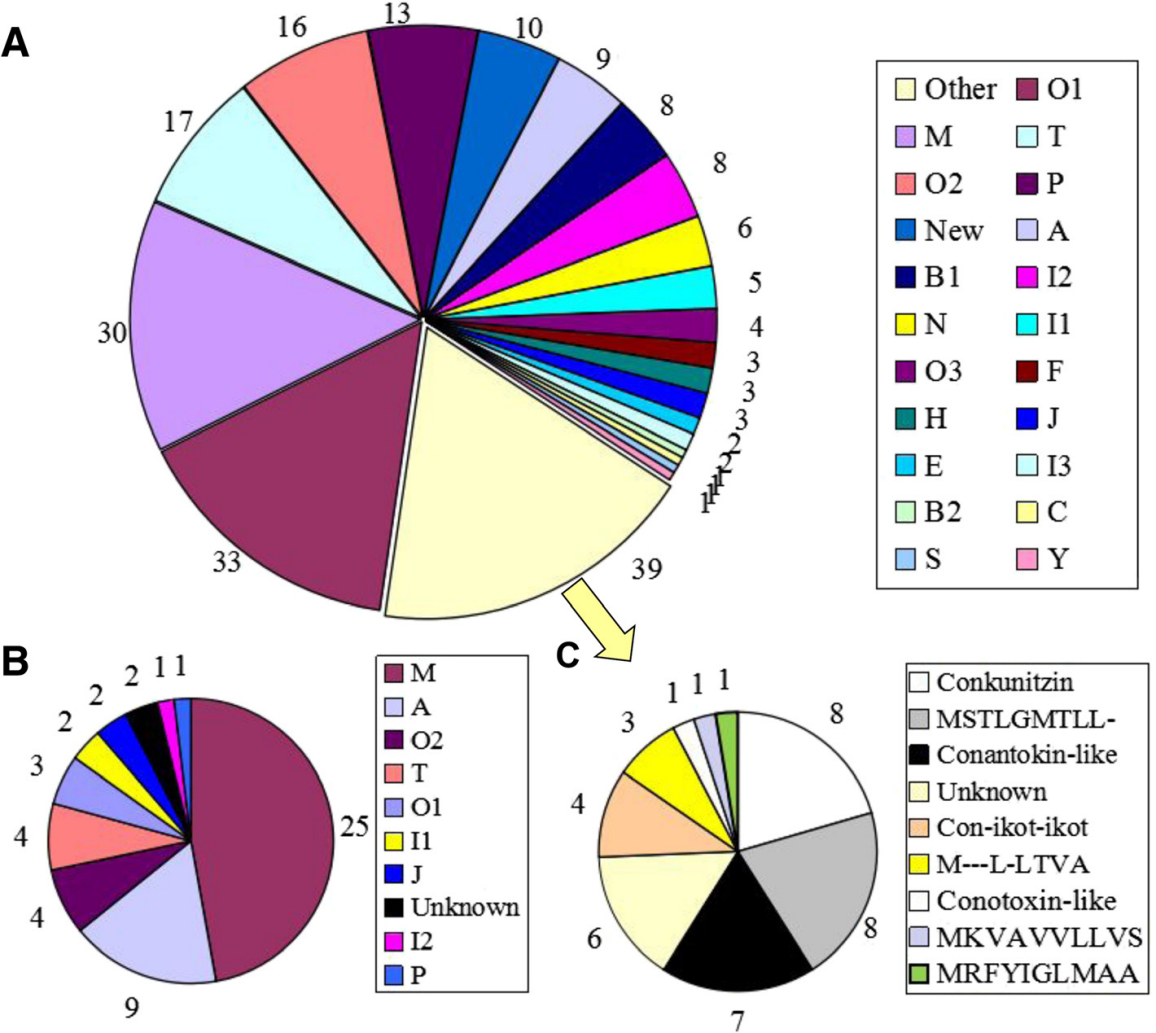
Summary of conopeptides in *C. betulinus*. **a** Total superfamilies or groups of conopeptides that were identified in this study. **b** The conopeptides that were reported previously. **c** Subdivision of the conopeptides from the ‘Other’ group in (**a**) into further categories, listed in decreasing order of frequency

To date, most studies have specifically focused on the piscivorous and molluscivorous cone snails, whereas there is still relatively little research on the abundant vermivorous species (which account for about 75 % of all cone snails) [40, 58, 63, 64]. As a worm-hunting species, the Chinese tubular cone snail (*C. betulinus* (Linnaeus)) is a dominant *Conus* species inhabiting the South China Sea. In previous works on this species [42], only 53 mature conotoxins from nine gene superfamilies (Fig. 1b) were derived from precursors or via traditional approaches (see Additional file 1). Next-generation whole-transcriptome sequencing of the *C. betulinus* venom duct has never been attempted. Our current study therefore surveyed conotoxin cDNA precursors using a variety of strategies, including sampling individuals with different body sizes, sampling different tissues, preparing samples with different normalization strategies, and employing different sequencing methodologies. This resulted in six datasets (see Methods for details). Body sizes were categorized as Big, Middle and Small: the Big specimen was 10 cm in body length, the Middle one was 8.7 cm and the Small specimen was 6 cm.

A summary of five transcriptome assemblies (excepting the expressed sequence tag (EST) dataset) is presented in Table 1. The Illumina sequencing generated 5.46, 4.58, 8.39, 4.67 and 9.77 Gb of raw sequences in the datasets Normalized, Small, Middle, Big and Bulb, respectively (see more details in Methods). After trimming low-quality reads, 5.23, 4.37, 7.95, 4.42 and 9.16 Gb of corresponding clean reads were obtained and used for subsequent assembly. Using the *de novo* transcriptome assemblers Trinity and SOAPdenovo-Trans [65, 66], the clean reads from the five datasets were separately assembled into contigs. For improved assembly quality, a clustering step was performed by eliminating redundant contigs [67]. Contigs were then further assembled into between 136,569 and 180,492 unique transcripts with a mean length of 394 to 544 bp and an N50 length of 398 to 681 bp for the five transcriptomes.

**Table 1:** Summary of the transcriptome assemblies

		Venom Duct				Venom
		Normalized	Small	Middle	Big	Bulb
Total raw reads		60,707,418	50,926,032	93,247,084	51,896,884	108,589,106
Total raw nucleotides (nt)		5,463,667,620	4,583,342,880	8,392,237,560	4,670,719,560	9,773,019,540
Total clean reads		58,091,534	48,557,542	88,318,918	49,161,726	101,724,490
Total clean nucleotides (nt)		5,228,238,060	4,370,178,780	7,948,702,620	4,424,555,340	9,155,204,100
Unique gene	Number	87,714	114,057	52,387	94,026	124,004
	Total length	45,438,256	44,918,779	23,128,493	37,880,261	67,451,577
	Max length	16,974	14,747	12,264	9,564	18,070
	Mean length	518	394	441	403	544
	N50	612	398	464	413	681

*The Normalized transcriptome was assembled by SOAPdenovo-Trans 1.02 and the transcriptomes of Big, Middle, Small and Bulb were assembled using Trinity software. Evaluation of two assemblers for the Normalized sample showed essentially equal performance.

In parallel, a cDNA library, generated from a pool of total RNA from the venom ducts of six specimens, was sequenced by using an ABI 3730 (Sanger-type). After removing vector sequences, primer sequences and poly(A) tails, 11,026 clean ESTs were obtained, with an average length of 663 bp. Redundancy among these ESTs was further eliminated and a total of 5798 unique transcripts, averaging 692 bp in length, were finally acquired.

## Results

### Total conopeptides identified in the current study

The putative conopeptide sequences were predicted by BLASTX search and HMMER analysis [68] (Additional files 2 and 3) against a local reference database of known conopeptides from the ConoServer databases [41], and then examined manually using the ConoPrec tool [42]. After removal of the transcripts with duplication or truncated mature region sequences, we obtained totals of 46, 123, 98, 94, 95 and 39 putative conopeptide transcripts for the six datasets of EST, Normalized, Small, Middle, Big (non-normalized venom ducts) and (venom) Bulb, respectively. The majority of these identified conopeptides were full-length or nearly full-length, although a few conopeptides contained mature region sequences only. For these partial sequences, we tried to derive the missing regions with RT-PCR and Sanger sequencing.

We combined the six conopeptide datasets into a ‘Total conopeptide dataset’ and named the 215 putative conopeptides identified as Bt001 to Bt215 (Additional file 4). They each have at least one amino acid (aa) difference from one another in the mature regions. Among these 215 conopeptides, 178 were classified into 20 previously reported superfamilies (A, B1, B2, C, E, F, H, I1, I2, I3, J, M, N, O1, O2, O3, P, S, T and Y; Fig. 1a) and two cysteine-rich families (like Conkunitzin and Con-ikot-ikot; Fig. 1c). Seven of the conopeptides were highly similar to the ‘conantokin-like’ group (Fig. 1c), which belongs to the peculiar cysteine-poor Conantokin family but is not classified with the B1 superfamily in the ConoServer database because of the obvious difference in the signal region sequences.

**Fig. 2:**
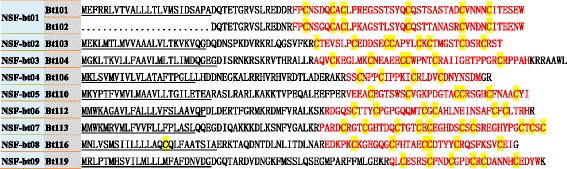
New superfamilies of conopeptides identified in *C. betulinus*. The ten conopeptides in the ‘New’ group of Fig. 1a have been clustered into nine new superfamilies (designated NSF-btXX), according to their signal peptide sequences. The signal regions predicted by the ConoPrec tool are underlined, and the mature regions (shown in red) and cysteine residues (highlighted yellow) are marked for comparison

In addition, the sequence identities in the signal regions of ten putative conopeptides (Fig. 2) in the ‘Total conopeptide dataset’ were below the threshold values for any empirical superfamilies (see more details in Methods). Therefore, the ConoPrec tool [42] was used to analyze these ten conopeptide precursors and identify their signal sequences. It was confirmed that the majority of these conopeptides (Bt101, Bt103, Bt110 and Bt113) contain three common regions, i.e. signal region, pro-region and mature region. However, several conopeptides, including Bt104, Bt106, Bt112, Bt116 and Bt119, have a short peptide sequence after the mature region, and Bt102 contains only partial pro- and mature regions. Finally, all these novel putative conopeptides were classified into nine new conopeptide superfamilies (Fig. 2), designated as NSF-bt01 to NSF-bt09.

Among the 53 previously published conopeptide sequences of *C. betulinus* (Additional file 1), 26 were recovered in our ‘Total conopeptide dataset’ (Fig. 1b and Additional file 1). Half of these 26 conopeptides belong to the M-superfamily, which accounts for 52 % of the M-conotoxins published previously for this species. Four conotoxins of the T-superfamily have been reported in *C. betulinus* before, and all of these were re-identified in our current study. Some published conopeptides from this species, belonging to the A, I1, I2, O1 and O2 superfamilies, were also confirmed, whereas none belonged to the J and P superfamilies (Additional file 1).

**Fig. 3:**
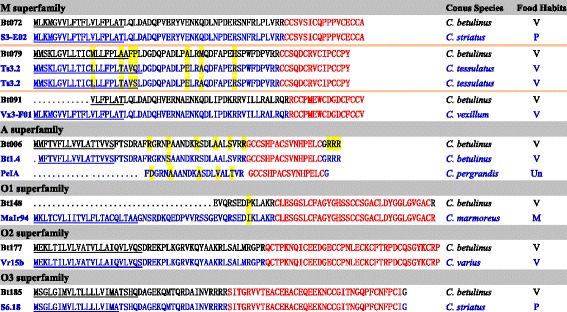
Comparison of seven *C. betulinus* conopeptides with their homologous sequences reported from other *Conus* species. The conopeptides identified in our current study are shown in black, and the reference sequences are marked in blue. Note that the mature regions (shown in red) are highly conserved. The names of the reference sequences are derived from the ConoServer database. The signal regions are underlined, and the dissimilar residues are highlighted in yellow. (Food habits: M, molluscivorous; P, piscivorous; Un, undescribed; V, vermivorous)

Interestingly, only seven of the 215 total conopeptides have the same mature region sequences as previously reported in other cone snail species (Fig. 3 and Table 2). Among these, three conopeptides (Bt072, Bt079 and Bt091) belong to the M-superfamily, and the remaining four (Bt006, Bt148, Bt177 and Bt185) are classified into the A, O1, O2 and O3 superfamilies, respectively. There is no difference in their precursor sequences between Bt072 and the reported conotoxin S3-E02 of piscivorous *C. striatus*. The same situation also occurs between Bt185 and S6.18 (*C. striatus*), and between Bt177 and Vr15b (*C. varius*, vermivorous). Two precursor sequences of Ts3.2 from the vermivorous *C. tessulatus* have only one aa difference in the signal region; our newly identified Bt079 has exactly the same mature region, but has four and three aa differences in the signal sequences and pro-regions, respectively. Bt091 has apparently lost part of its signal region, but its remaining sequences are consistent with Vx3-F01 from the vermivorous *C. vexillum*. Despite missing its signal sequence and partial pro-region, Bt148 showed an identical mature region and almost the same pro-region (only one aa mutation) as MaIr94 of the molluscivorous *C. marmoreus*. The mature peptide sequence of Bt006 had been reported in both *C. betulinus* (named as Bt1.4) and *C. pergrandis* (PeIA) [69, 70]; however, there is a one-aa difference in the pro-region between Bt006 and Bt1.4, and PeIA has more aa mutations (nine) in the pro-region when compared with Bt006 and Bt1.4.

**Table 2:** Expression levels of the seven conotoxins covered in Fig. 3

Conopeptide	Superfamily	Small		Middle		Big		Reference conotoxin^a^
		RPKM	Ranking	RPKM	Ranking	RPKM	Ranking	
Bt072	M	3,110	19/98	25,964	8/94	—	—	S3-E02
Bt079		—	—	—	—	1331	38/96	Ts3.2
Bt091		19	85/98	—	—	—	—	Vx3-F01
Bt006	A	1,380	33/98	—	—	—	—	PeIA/Bt1.4
Bt148	O1	—	—	—	—	3034	24/96	MaIr94
Bt177	O2	437	51/98	649	55/94	628	52/96	Vr15b
Bt185	O3	—	—	17,584	13/94	—	—	S6.18

^a^Names of the reference conotoxins are derived from the ConoServer database. — indicates undetectable

These seven conotoxins demonstrated significant differential expression during the growth of *C. betulinus* (Table 2). For example, Bt079 and Bt148 were only present in the Big dataset, Bt006 and Bt091 were only expressed in the Small dataset, and Bt185 was expressed only in the Middle dataset; Bt072 was expressed in both the Small and Middle datasets, but was absent from the Big dataset; Bt177 was the only conotoxin identified in all three of the datasets from differently sized snails.

### Comparison of conopeptides in the three venom duct transcriptomes

**Fig. 4:**
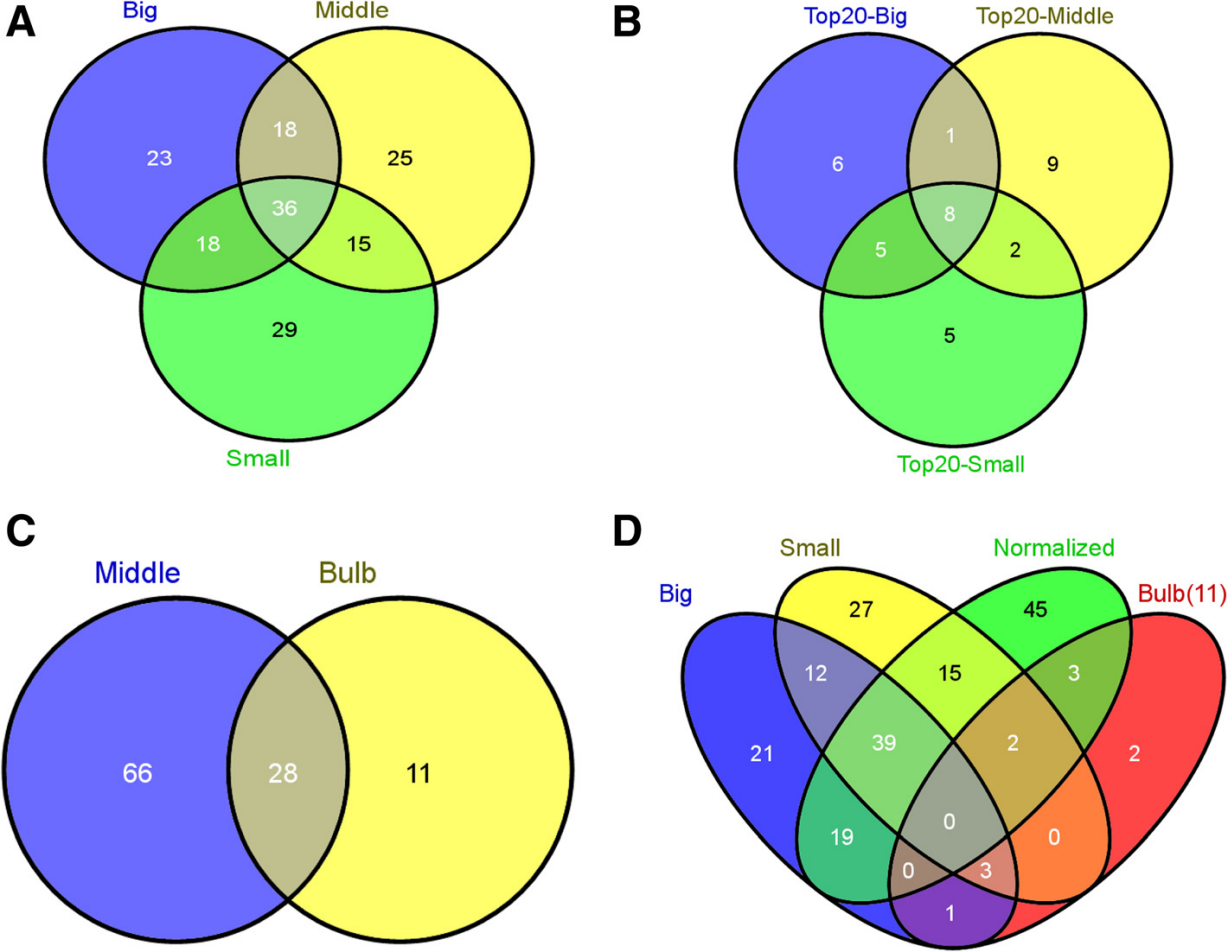
Venn diagrams of conopeptide transcripts from various *C. betulinus* datasets. **a** Relationship of the identified conopeptides from the Small, Middle and Big datasets. **b** The top 20 conopeptide transcripts (with the highest RPKM values) from the three datasets are compared with each other. **c** Comparison of total conopeptides from the ‘Middle’ venom duct and venom bulb datasets of the same Middle-sized specimen. **d** Comparison of the conopeptides from the other three venom duct datasets with the 11 putative bulb-specific transcripts identified in (**c**), to reveal two potential venom bulb-specific conotoxins (in area colored red)

A total of 98, 94 and 95 putative conopeptide precursors, respectively, were identified from the Small, Middle and Big datasets from the venom ducts of three body-sized *C. betulinus*. The comparative distribution of conopeptides is summarized in the Venn diagrams of Fig. 4a,b. The 36 common conopeptides were classified into 21 superfamilies, of which 17 were described in the ConoServer database and three were new superfamilies. Most of these identified peptides belong to the O1 and M superfamilies and the Conkunitzin group. Around the same number of unique conopeptides were present together within each pairing of the three transcriptomes (51, 54 and 54 common precursors in the Small & Middle, Middle & Big, and Big & Small, respectively). In the same way, the number of conopeptides identified as specific to any one of the three body-sized specimens was similar (29, 25 and 23 from the Small, Middle and Big datasets, respectively).

**Fig. 5:**
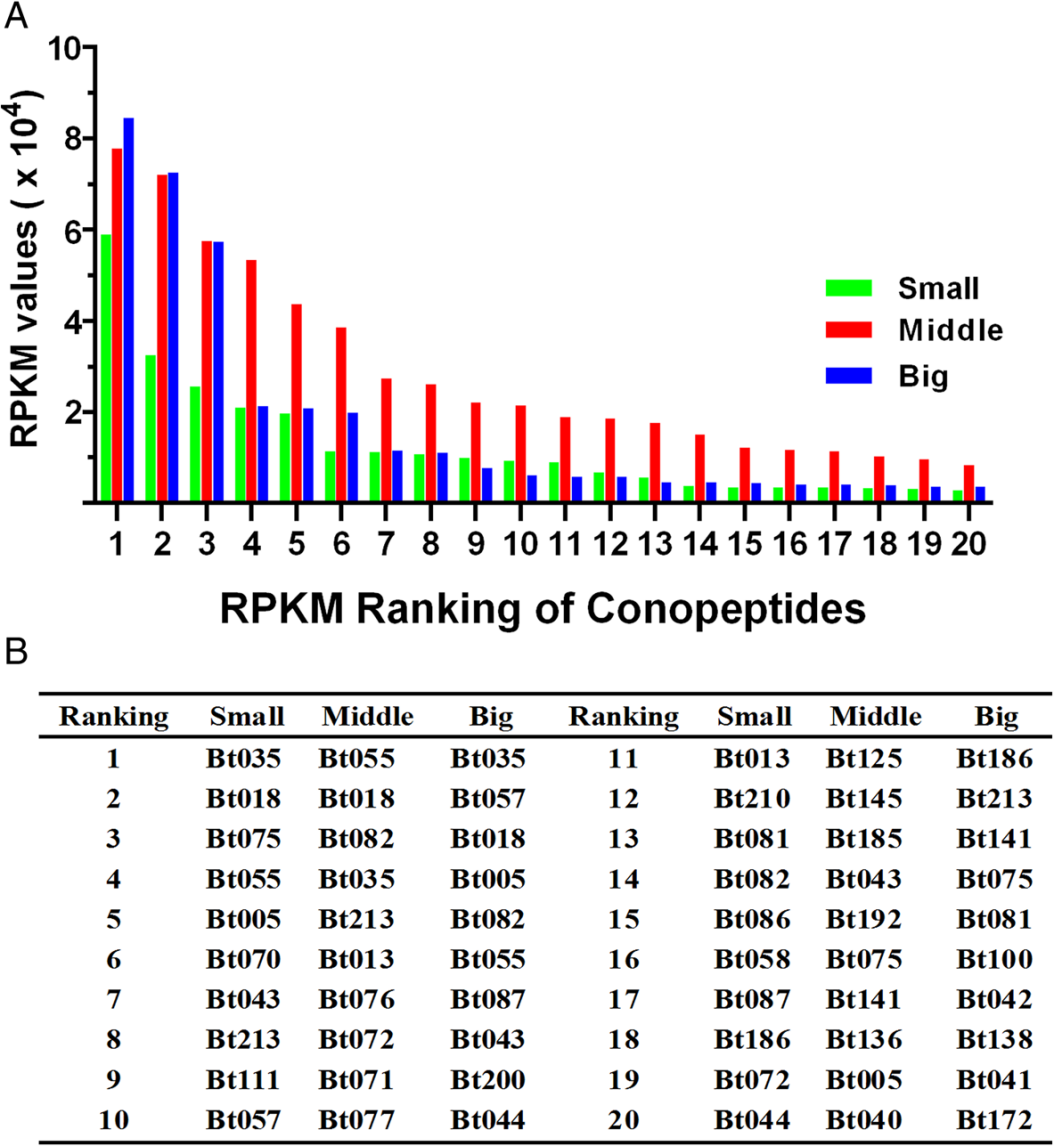
The top 20 conopeptides (with the highest RPKM values) from three transcriptomes. **a** Comparison of the top 20 conopeptides from each of the Small, Middle and Big datasets. **b** The RPKM ranking of individual conopeptides within each of the datasets

RPKM (reads per kilobase of transcript per million mapped reads) values were calculated to represent the expression levels of each conopeptide. The top 20 conopeptides (with the highest RPKM values) were selected from each of the datasets, and it was found that expression levels in the Middle snail were generally higher than those in the Small or Big specimens. The top 18 conopeptides in the Middle specimen, as well as the top eight conopeptides in both the Small and Big specimens, had RPKM values above 10,000 (Fig. 5). Eight transcripts were common to the top 20 conopeptides of the three datasets (Fig. 4b), but their RPKM ranking was variable (Table 3). For example, Bt035, the highest-ranked in both the Small and the Big specimens, ranked only fourth in the Middle snails. Using the National Center for Biotechnology Information’s (NCBI) BLASTP tool, we also found that Bt035 is similar to the Eb-conantokin-like protein from *C. eburneus* and the conantokin-F peptide from *C. flavidus* in its precursor sequences (73 and 65 % identity, respectively), suggesting Bt035’s conantokin-like status and potential neuronal NMDAR-inhibiting activity. Interestingly, Bt018, with high expression levels in all three specimens (Table 3), may be the first B2-superfamily conopeptide identified in *C. betulinus*, because its signal sequence possesses high similarity (only one aa substitution) to this superfamily.

**Table 3:** The conopeptides that are common among the three top 20s

Conopeptide	Ranking of RPKM			Superfamily	Possible activity^a^
	Small	Middle	Big		
Bt035	1	4	1	Conantokin-like	NMDA receptor inhibitors
Bt018	2	2	3	B2	unknown
Bt075	3	16	14	M	α,ι,κ,μ
Bt055	4	1	6	I2	κ
Bt005	5	19	4	A	α
Bt043	7	14	8	H	δ,γ,κ,μ,ω
Bt213	8	5	12	T	ε,μ,τ
Bt082	14	3	5	M	unknown

^a^The Greek letters denote pharmacological families defined in ConoServer as conopeptides sharing the same receptor specificities

Bt075 and Bt082 (a conomarphin) belong to the M-superfamily. Bt075 is identical to the Bt3-3-VP02 conotoxin previously reported in *C. betulinus* [71]. However, Bt082 contains the cleavage site KR, producing a mature 17-aa linear peptide with no cysteine framework, which is different from the more common 15-aa mature peptide in previously examined conomarphins (Conomarphin-Bt1, 2 and 3).

Bt055, a representative of the I2-superfamily, was the highest-ranked conopeptide in the Middle specimen; its mature region sequence matches the cysteine framework of the previously identified kappa-Btx (Additional file 1). The A-superfamily Bt005 is highly similar to both the α-conotoxin-like Lp1.7 from *C. leopardus* [72] and Lt1c from *C. litteratus* [73].

There are only ten conopeptides classified into the H-superfamily in the ConoServer database, including seven from *C. marmoreus* [44] and three from *C. victoriae* [74]. Our Bt043 is the first H-superfamily conopeptide identified in *C. betulinus*, with high expression levels and a classical VI/VII cysteine framework. On the other hand, all the known T-superfamily conotoxins in *C. betulinus* belong to cysteine framework pattern V and have four cysteines; however, our Bt213 has the longest mature peptide sequence, with 23–33 aa more than any previously reported in the same species (Additional files 1 and 4).

### Differential expression of conopeptides in the venom duct and the venom bulb

**Fig. 6:**
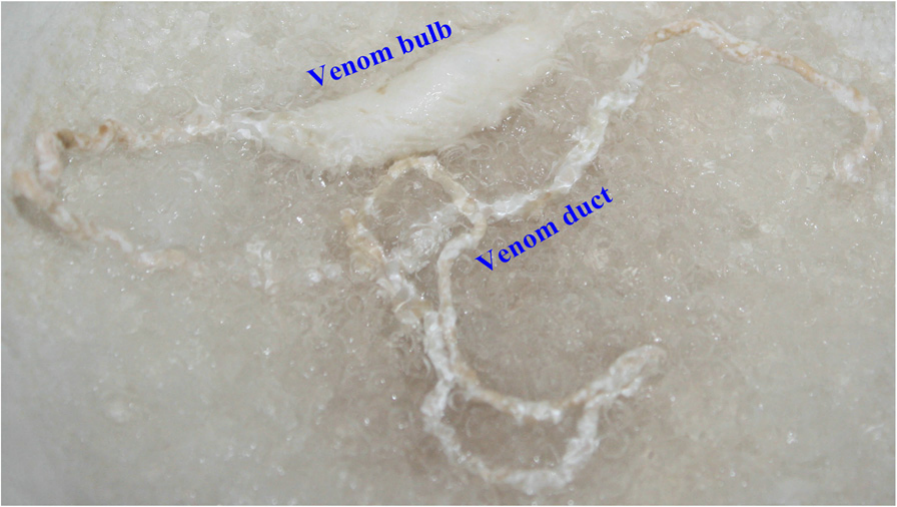
Dissection of the venom duct and the venom bulb in *C. betulinus*. Although the two regions are morphologically connected, our transcriptomic data demonstrated differential expression of conopeptides between them

**Fig. 7:**
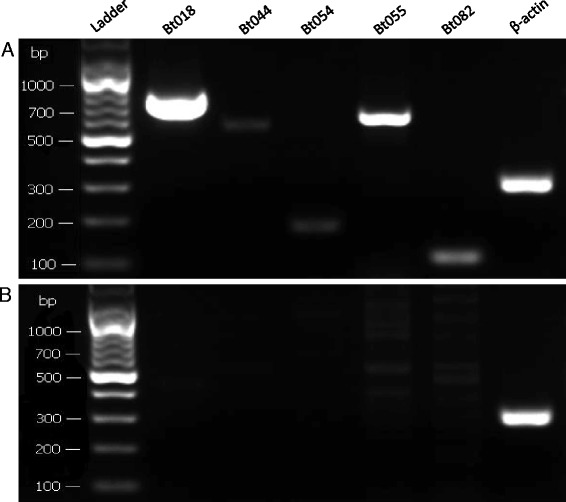
Confirmation by RT-PCR of expression differences of five randomly selected conopeptides. The PCR templates were from the venom duct (**a**) and the venom bulb (**b**). Beta-actin was used as the internal control

To determine if conopeptides are transcribed in the venom bulb, we dissected this tissue away from the venom duct of the Middle specimen of *C. betulinus* (Fig. 6) for further transcriptome sequencing. The total number of unique conopeptide sequences identified in the venom bulb (39) was less than half of the number identified in the venom duct (94). In the Bulb dataset, 16 known superfamilies, four new superfamilies and two groups of conopeptides were identified. Most of the conopeptides (17) belong to the M and O1 superfamilies and the Conkunitzin group. Sequences of the A, B, C, F, H, I, P and T superfamilies, as well as the conantokin-like group, were also observed. As expected, the expression levels of conopeptides identified in the venom bulb were far lower than those in the venom duct. The RPKM values of all conopeptides in the Bulb dataset were below 180; in contrast, in the Middle dataset, the highest and median RPKM values were 77,776 and 1021 respectively. We randomly picked several conopeptides (Bt018, Bt054, Bt055 and Bt082) and confirmed the differential expression between the venom bulb and the venom duct by RT-PCR (Fig. 7 and Additional file 5).

Between the two transcriptomes of venom duct and venom bulb from the Middle specimen, 28 conopeptides were revealed to be common, whereas 66 and 11 were unique to the venom duct and the venom bulb, respectively (Fig. 4c). Interestingly, among these 11 potential venom bulb-specific conopeptides, two M-superfamily members, Bt070 (Bt3-D05) and Bt089 (Bt3-TP04), had been identified in a previous study [71]; the other nine, belonging to nine different superfamilies or groups (B1, F, I1, O1, O2, O3, T, NSF-bt01 and Conkunitzin), were reported for the first time. When we compared the venom bulb dataset with all the datasets of the venom duct, nine of the sequences, including Bt070 (Bt3-D05) and Bt089 (Bt3-TP04), among these 11 potential unique conopeptides were revealed to be common to the two parts, whereas Bt048 and Bt168 remained unique to the venom bulb (Fig. 4d). However, there may have been a certain amount of contamination, since a tiny portion of the venom duct inside the venom bulb could not be removed and had to be considered as part of the venom bulb.

## Discussion

Sanger sequencing technology previously generated 19 conotoxin cDNA sequences of *C. striatus* and 42 of *C. litteratus* [73, 75]. In contrast, next-generation sequencing, a relatively inexpensive and efficient technology, has been applied recently to the venom duct transcriptomes of several *Conus* species [44, 53, 58, 59, 74, 76] and between 61 and 136 conopeptide sequences, belonging to 11–30 superfamilies, were discovered. To investigate the diversity of conopeptides in a single species using high-throughput methodologies, we applied both traditional Sanger sequencing and the next-generation Illumina Hiseq2000 sequencing platforms to study the venom duct and the venom bulb transcriptomes of *C. betulinus*.

**Fig. 8:**
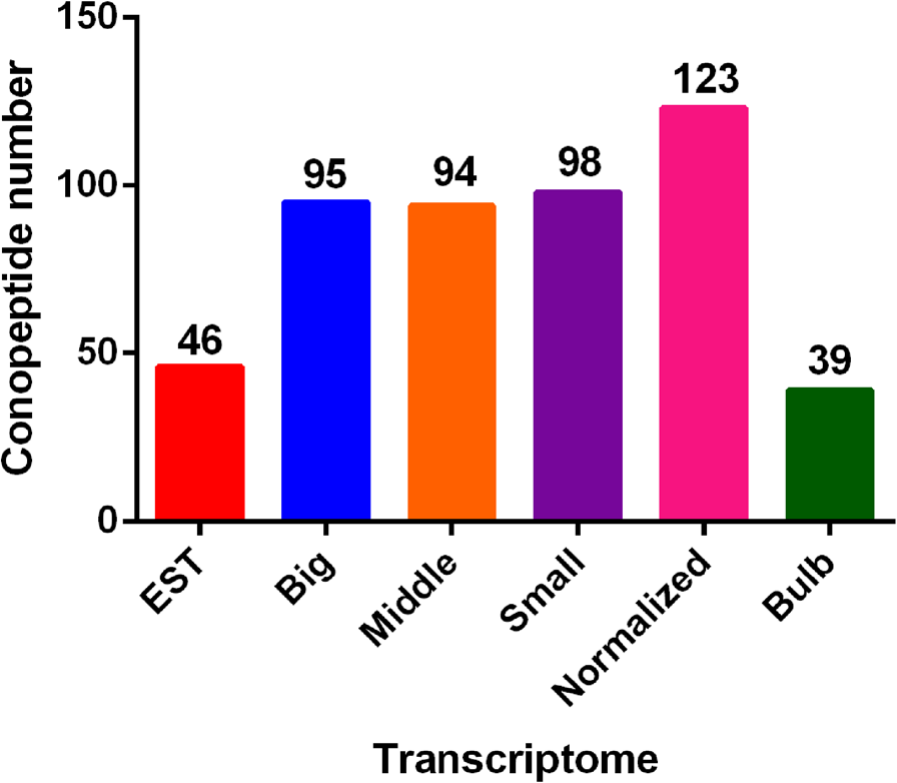
Number of conopeptides identified from different transcriptomes. The differences could be the result of differential sequencing method, sample preparation method, and/or specimen and tissue differences. The Normalized transcriptome was assembled by SOAPdenovo-Trans 1.02 and the transcriptomes of Big, Middle, Small and Bulb were assembled using Trinity software. Evaluation of the two assemblers for the Normalized sample showed essentially equal performance.

We report here over 200 conopeptide transcripts in this one *Conus* species. Based on traditional large-scale cloning of cDNA libraries and Sanger sequencing, only 46 conotoxin transcripts were detected from a mixed library of venom duct mRNAs. However, the number of conopeptide sequences identified from three non-normalized venom duct transcriptomes (from Small, Middle and Big specimens) and normalized venom duct/bulb transcriptomes (from the Middle specimen) was as high as 123 in each dataset with next-generation sequencing (Fig. 8). The dramatic difference between the two approaches once again demonstrates the value of this new transcriptome sequencing technology as a high-throughput method for discovering novel conotoxin genes. In addition, normalization during sample preparation can significantly increase the total conopeptide numbers compared to non-normalized libraries (from 94–98 up to 123; Fig. 8) but, as found here, additional non-overlap of transcripts was detected (Fig. 4d).

Our transcriptome of the venom bulb from *C. betulinus* demonstrates the presence of conotoxins in this tissue. Although only 39 conotoxin transcripts with very low expression levels were confirmed by transcriptome sequencing and RT-PCR, at least two of them (Bt048 and Bt168) were not identified from any venom duct dataset, which indicates they may be unique to the venom bulb. This provides confirmatory evidence for previous work comparing the venom duct and the venom bulb in which one-dimensional gel electrophoresis and RT-PCR showed expression levels (albeit low) of several proteins important for toxin biosynthesis, suggesting that the venom bulb engages in conotoxin biosynthesis [51].

After removal of duplications from the five transcriptomes and one EST sequencing dataset (Fig. 8), we obtained a total of 215 unique putative conopeptide transcript sequences, which were classified into 37 superfamilies and groups with at least one aa difference in the mature regions between each other. This represents a very high number of novel conopeptide transcripts discovered from a single species of *Conus*.

**Fig. 9:**
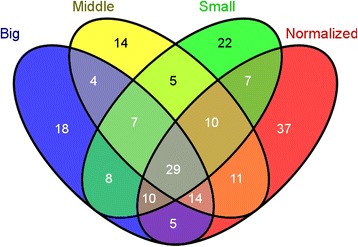
Comparison of conopeptides from the four venom duct transcriptomes. Only 29 conopeptides are common among the four transcriptome datasets (in area colored gray)

Previous studies have shown that venom composition varied dramatically even among individuals from the same species, and suggested the presence of intraspecific variability in the *Conus* venom peptides [51, 55, 77–81]. However, a recent report by Dutertre *et al.* [44] revealed that thousands of conotoxins may be derived from only hundreds of conopeptide genes. Our comparison of the venom duct transcriptomes of three body-sized *C. betulinus* (the Small, Middle and Big datasets) has authenticated the presence of 28 to 32 gene superfamilies in each individual. By comparing each pair of the three transcriptomes, we found that the number of common conopeptides ranged from 51 to 54 and the individual-specific conopeptides averaged 42 (40–47; Fig. 4a). A wider comparison, in Fig. 9, reduced the individual-specific numbers to 22, 14 and 18, respectively. Hence, it is likely to be possible to identify more novel conotoxins via the sequencing and comparison of additional specimens, and the total number of conopeptide genes existing in a species may be comfortably above early estimates that ranged from 50 to 200 conopeptides per *Conus* species. The exact numbers can be confirmed by whole genome sequencing, which is underway for *C. betulinus* in our laboratories.

## Conclusions

This study is the first report to examine the diverse conopeptide expression repertoire in the vermivorous Chinese tubular cone snail, *Conus betulinus*. We used multi-transcriptome sequencing and complemented by traditional Sanger sequencing and a total of 215 unique putative conopeptide sequences were identified. As anticipated, most (183) of the identified conopeptides were novel, and nine new superfamilies were classified. We also demonstrated the differential expression of conopeptide genes among three individuals with different body sizes (at potentially different developmental stages), and our data suggest the existence of remarkable intraspecific variability in the venom duct. It is therefore probable that the discovery of more novel conotoxins will be accomplished via sequencing and analyzing additional specimens. Meanwhile, comparison of conopeptide expression in the venom duct and the venom bulb sheds light on the presence of conotoxins outside the venom duct. We demonstrated for the first time the existence of a handful of conotoxins in the venom bulb, although their expression levels were relatively low.

## Methods

### Sample collection and RNA extraction

Eight specimens of *Conus betulinus* (Additional file 6), 6–10 cm in length, were collected in the offshore areas of Sanya City, Hainan Province, China. Immediately after collection, the snails were placed on ice and dissected. Taxonomic identification was confirmed by COI sequences (DNA barcoding), which are identical to the reported data (GenBank accession numbers HQ834088.1, KJ549869.1, JN053043.1 and JF823627.1). Total RNA was extracted from the venom ducts of all specimens and the venom bulb of one middle-sized snail using TRIzol® LS Reagent (Invitrogen, Life Technologies, USA) according to the manufacturer’s instructions. Isolation of mRNA molecules containing poly(A) tails was carried out via the use of oligo-(dT)-attached magnetic beads (Invitrogen). All experiments were performed in accordance with the guidelines of the Animal Ethics Committee and were approved by the Institutional Review Board on Bioethics and Biosafety of BGI (No. FT15103).

### Construction and sequencing of cDNA libraries

To maximize the numbers of conopeptides identified from the specimens, three methods were applied to construct different cDNA libraries. The first was to construct a full-length cDNA library of mixed mRNAs from the venom ducts of six snails of various sizes, and around 11,000 clones were sequenced from 5’ to 3’ using an automated ABI 3730 sequencer. The second approach was to choose a medium-sized specimen for construction of a normalized Illumina cDNA library. cDNAs were normalized using a duplex-specific nuclease (DSN) approach according to the DSN Normalization Sample Preparation Guide (Early Access Protocol, Part number 15014673 Rev. C, Illumina, 2010). Third, and most importantly, we constructed four non-normalized Illumina cDNA libraries using mRNAs from, respectively, the venom ducts of three snails with different body length and body weight, and a venom bulb from the middle-sized specimen. For nomenclature, the four non-normalized transcriptome datasets were named ‘Big’ (venom duct of a snail 10 cm in body length), ‘Middle’ (venom duct of a snail 8.7 cm in length), ‘Small’ (venom duct of a snail 6 cm in length) and ‘Bulb’ (venom bulb from the middle-sized snail). In addition, a normalized transcriptome of another medium-sized snail was referred to as the ‘Normalized’ dataset. The traditional transcriptome of cDNA libraries (reverse transcribed from total mRNAs, cloned, and sequenced by ABI 3730 Sanger methodology) was called the ‘EST’ dataset.

### Sequence data processing (analysis and assembly)

In order to obtain high-quality clean reads for *de novo* assembly, the raw reads generated from transcriptome sequencing were filtered with the following steps: (1) adaptor sequences were removed; (2) reads with more than 10 % of unknown nucleotides were removed; (3) reads with more than 50 % of low-quality bases (base quality ≤10) were discarded. The clean reads that remained were assembled into unique genes using Trinity software [65] with an optimized k-mer length of 25 for *de novo* assembly, except for the Normalized transcriptome of venom duct that was assembled by SOAPdenovo-Trans 1.02 [66]. The expression of unique genes was calculated using RPKM, which is a general method of quantifying gene expression from RNA sequencing data by normalizing for total read length and the number of sequencing reads [82]. We represent the expression of each unique transcript using RPKM values, instead of sequencing depth/coverage, because the values are normalized and facilitate comparisons.

Meanwhile, raw sequences of the EST dataset from the ABI 3730 sequencing were trimmed by removal of vector sequences, primer sequences, and poly(A) tails with ABI Prism DNA Sequencing Analysis v5.4 software to obtain high-quality clean EST sequences. Redundant sequences were removed from the dataset.

### Prediction and identification of conopeptides

We applied homology searches and an *ab initio* prediction method [83] to predict conotoxins from the five transcriptomes and one EST sequencing dataset.

For homology searches, all previously known conopeptides were downloaded from the ConoServer database [42] to construct a local reference dataset. We subsequently used BLASTX (with an E-value of 1e-5) to run our assembled sequences against the local dataset. Those unique genes/ESTs with the best hits in the BLASTX data were translated into peptide sequences.

In addition, an *ab initio* prediction method using a Hidden Markov Model (HMM) was adopted to discover new conopeptides. First, the reference dataset of known conopeptides from the ConoServer database [42] was grouped into different classes according to their published superfamily and family classifications. Second, sequences of each class were aligned with the ClustalW tool [84] and the ambiguous results were checked using the ConoPrec tool [42] and manual inspection. Finally, a profile HMM was built for the conserved-domain of each class using *hmmbuild* from the HMMER 3.0 package [68] to find the best HMM parameter, and the *hmmsearch* tool was then applied, using this trained HMM parameter, to scan every unique assembled gene/EST for identification of conopeptides.

### Classification of gene superfamilies

The predicted conotoxin transcripts were manually inspected using the ConoPrec tool implemented in the ConoServer database [42]. Those transcripts with duplication or truncated mature region sequences were removed. The signal peptides, gene superfamilies and cysteine frameworks of these predicted conopeptides were also checked for confirmation. Based on 75 % identity in the highly conserved signal peptide sequences [40], the conopeptide precursors could be assigned to most of the gene superfamilies present in the ConoServer database. Particular cut-off values were then used for some gene superfamilies with lower conservation of the signal region. The threshold values for assigning the conopeptides to I1, I2, L, M, P, S, con-ikot-ikot and divergent superfamilies were adjusted to 71.85, 57.6, 67.5, 69.3, 69.1, 72.9, 64.5 ± 20.2 and 64.22 ± 20.53 %, respectively [76]. If the conservation of a signal region was below the threshold value for any reported conotoxin superfamily, the conopeptide was regarded as a member of a new gene superfamily named in the form ‘NSF-bt’ plus an Arabic number suffix. Those conopeptides without signal regions but still showing similarity either in the pro- or mature region were considered as an ‘Unknown’ group.

### Reverse transcription PCR

After extraction of total RNA from the venom duct and venom bulb of the Middle specimen, cDNAs were reverse transcribed using the M-MuLV First Strand cDNA Synthesis Kit (Sangon, China). We randomly selected five conopeptides and employed Primer Premier 5.0 to design primer pairs (see detailed nucleotide sequences in Additional file 5). Reverse transcription PCR (RT-PCR) was performed in 50-μl reactions, containing 0.5 μl of cDNA, 0.5 μl of rTaq DNA Polymerase (Takara, Japan), 1 × PCR reaction buffer (Takara), 200 μM of each dNTP, and 0.2 μM of forward and reverse primers. The targeted DNAs were amplified in an ABI 9700 thermal cycler (Life Technologies, USA) as follows: initial denaturation at 95 °C for 5 min; 35 cycles of 94 °C for 30 s, 55 °C for 30 s and 72 °C for 45 s; final extension at 72 °C for 10 min. Beta-actin was used as an internal control. All the PCR amplicons were checked by 1.5 % agarose gel electrophoresis for comparison of relative expression levels.

### Availability of supporting data

The datasets supporting the results of this article are included within the article and its additional files. The transcriptome reads produced in this study have been deposited in the NCBI SRA database with accession numbers SRS1009725 for the Big dataset, SRS1009729 for the Middle dataset, SRS1009726 for the Small dataset, SRS1009727 for the Normalized dataset, and SRS1009728 for the Bulb dataset. The clean reads for 11,026 clones were sequenced by ABI 3730 and submitted to the NCBI as EST data (PRJNA290540). Additional file 7 provides the translated sequences of conopeptides identified from the EST dataset; all of the 215 transcripts identified have been submitted to GenBank (accession numbers are included in Additional file 8). Additional supporting data is also hosted in the *GigaScience* GigaDB repository [85], as well as linked to NCBI bioproject number PRJNA290540.

## Additional files

Additional file 1: Summary of previously reported conopeptides in *C. betulinus*. (DOC 102 kb) Additional file 2: The alignment file used to generate the profile HMM (pHMM). (RAR 10 kb) Additional file 3: The actual pHMM file created for identifying conopeptides. (RAR 225 kb) Additional file 4: The FASTA file for the 215 conopeptides identified by us from *C. betulinus*. (TXT 17 kb) Additional file 5: Primer sequences used for RT-PCR (5’ to 3’). (DOCX 13 kb) Additional file 6: A picture of *Conus betulinus* (Linnaeus) native to the South China Sea. (JPG 4261 kb) Additional file 7: List of conopeptide coding sequences identified in the EST dataset. (XLSX 15 kb) Additional file 8: Accession numbers of the 215 identified conopeptides in the NCBI GenBank. (XLSX 15 kb)
