# Supplementary material for: High-throughput identification of novel conotoxins from the Chinese tubular cone snail (Conus betulinus) by multi-transcriptome sequencing
Source: Gigascience. 2016 Apr 14;5:17. doi: 10.1186/s13742-016-0122-9 (PMC4832519; doi:10.1186/s13742-016-0122-9)
Supplement: Additional file 1: — Summary of previously reported conopeptides in C. betulinus. (DOC 102 kb) [file 13742_2016_122_MOESM1_ESM.doc]

***Additional file 1: Summary of previously reported conopeptides in C.*** betulinus

| **Superfamily^a^** | **Trans^b^** | **Conopeptide^c^** | **Mature Sequence^d^** | **Family^e^** | **Activity** |
| --- | --- | --- | --- | --- | --- |
| A(9) | √ | Bt1.1 | SAT**CC**NYPP**C**YETYPES**C**L |  |  |
|  | × | Bt1.2 | GG**CC**SHPA**C**AVNHPEL**C** |  |  |
|  | × | Bt1.3 | IAMAISSGA**CC**AYPP**C**FEAYPER**C**L |  |  |
|  | √ | Bt1.4 | G**CC**SHPA**C**SVNHPEL**C** | α | nAChR |
|  | √ | Bt1.5 | NAE**CC**YYPP**C**YEAYPEI**C**L |  |  |
|  | × | Bt1.6 | NGR**CC**HPA**C**GKHFS**C** |  |  |
|  | × | Bt1.61 | AIKLT**CC**SELT**C**AGNYPNI**C** |  |  |
|  | √ | Bt1.7 | GG**CC**SYPA**C**SVEHQDL**C**D |  |  |
|  | × | Bt1.91 | K**CC**SNPA**C**NRYNPAI**C**D |  |  |
| I1(2) | √ | Bt11.1 | M**C**LSLGQR**C**ERHSN**CC**GYL**CC**FYDK**C**VVTAIG**C**GHY |  |  |
|  | × | Bt11.4 | M**C**LSLGQR**C**GRHSN**CC**GYL**CC**FYDK**C**VVTAIG**C**GHY |  |  |
| I2(1) | √ | BtX/BeTX | **C**RA(Gla)GTY**C**(Gla)NDSQ**CC**LN(Gla)**CC**WGG**C**GHO**C**RHP(nh2) | κ | VGKC(blocker) |
| J(2) | × | Bt14.1 | **C**PEM**C**NEGSGGVA**C**S**C**SKRRDVVSSFV |  |  |
|  | × | Bt14.3 | APAELILETI**C**PHM**C**GTGIGEPF**C**N**C**RNKRDVVSSRII |  |  |
| M(25) | √ | BeTXIb/BtIIIB | **CC**ELP**C**HG**C**VP**CC**WP |  |  |
|  | √ | Bt14-H01/Bt14-H02 | VW**C**DWEW**C**YGD**C**H**C**FD |  |  |
|  | × | Bt3.1 | **CC**HAPY**C**TPPHLG**C**P**CC** |  |  |
|  | × | Bt3.2 | **CC**TQS**C**TT**C**FP**CC** |  |  |
|  | × | Bt3.3 | **CC**QAA**C**SPWL**C**LP**CC** |  |  |
|  | × | Bt3.4 | **CC**QAG**C**SRYM**C**LP**CC**Q |  |  |
|  | × | Bt3.5 | YWTRSA**CC**YIEEGEK**C**PAS**C**KL**CC** |  |  |
|  | √ | Bt3.6/Bt3-IP01 | **CC**SRN**C**AV**C**IP**CC**PNWPA |  |  |
|  | √ | Bt3-3-VP02 | **C**R**C**EQT**C**GT**C**VP**CC** |  |  |
|  | × | Bt3-D02/Bt3-D04 | E**CC**ELEW**C**DGA**C**D**CC**D |  |  |
|  | √ | Bt3-D05 | E**CC**EWEW**C**DGA**C**D**CC**N |  |  |
|  | √ | Bt3-F02 | Q**CC**TVEW**C**DTD**C**F**CC**A |  |  |
|  | √ | Bt3-I02 | R**CC**ISPA**C**HDE**C**I**CC**ID |  |  |
|  | √ | Bt3-I03 | R**CC**VHPA**C**HDD**C**I**CC**IT |  |  |
|  | × | Bt3-I04 | R**CC**VHPA**C**HDD**C**I**CC**ID |  |  |
|  | √ | Bt3-I05 | Q**CC**DWPW**C**DD**C**I**CC**D |  |  |
|  | × | Bt3-IP01 | **CC**SQD**C**RV**C**IP**CC**PH |  |  |
|  | √ | Bt3-MP02 | A**CC**EQS**C**TT**C**MP**CC** |  |  |
|  | √ | Bt3-T01 | K**CC**TMSV**C**QPPPV**C**T**CC**A |  |  |
|  | √ | Bt3-TP04 | **CC**KVL**C**ES**C**TP**CC** |  |  |
|  | × | Bt9.1 | **C**IVGTP**C**HV**C**RSQSKS**C**NGWLGKQGY**C**GY**C** |  |  |
|  | √ | BtIIIA/BeTXIa | **CC**KQS**C**TT**C**MP**CC**W |  |  |
|  | × | Conomarphin-Bt1 | GWVYHANPEANSWWT |  |  |
|  | × | Conomarphin-Bt2 | GWVYHAHPEPNSFWT |  |  |
|  | × | Conomarphin-Bt3 | GWVYHAHPDANSWWS |  |  |
| O1(3) | √ | BeB42 | **C**NDPGGS**C**TRHYH**CC**QLY**C**NKQESV**C**LENEPAF |  |  |
|  | × | Bt6.1 | A**C**AEFGHS**C**ISAT**CC**PGVT**C**VEIDEPV**C**LWD |  |  |
|  | × | Bt6.4 | **C**AGIGSF**C**GLPGLVD**CC**SGR**C**FIV**C**LP |  |  |
| O2(4) | √ | BeB54 | KSTAESWWEGE**C**KGWSVY**C**SWDWE**CC**SGE**C**TRYY**C**ELW |  |  |
|  | √ | Bt15a | Q**C**TPRNQR**C**EGDAE**CC**PNLV**C**K**C**FTRPD**C**QSGYK**C**DTS |  |  |
|  | × | Bt6.3 | KTTAESWWEGE**C**SGWSVY**C**TWDSE**CC**SGE**C**TRSY**C**ELW |  |  |
|  | × | Contryphan-Bt1 | G**C**PPGLW**C** |  |  |
| P(1) | × | BeTXIIb | G**C**GGV**C**AYGES**C**PSS**C**NT**C**YSAQ**C**TAQ |  |  |
| T(4) | √ | BeB34 | A**CC**PYEPS**CC**I |  |  |
|  | √ | Bt5.2 | HFYLRDEDWVD**CC**QMGD**CC**DE |  |  |
|  | √ | Bt5.3 | VADD**CC**VGKVGT**CC** |  |  |
|  | √ | Bt5.4 | DEDWVD**CC**HMPR**CC**VED |  |  |
| Unknown(2) | × | BeTXIIa | R**C**AHGTYYSNDSQQ**C**LLN**CC**WWGGGDH**CC**R |  |  |
|  | × | Bt6.2 | **C**SEVGAA**C**DTESNI**CC**SGE**C**FAVQGSTFGI**C**E |  |  |

^a^This number in parentheses is the previously known number of members of this Superfamily in *C. betulinus*. ^b^√means that the conopeptide was comfirmed in this work, and×means that not found in this work. ^c^Names of the reference conopeptides are derived from the ConoServer database. ^d^Cysteine residues are marked in bold, gamma carboxylic glutamic acids are represented as Gla, and hydroxyproline O- and C-term amidation was abbreviated as nh2. ^e^The pharmacological families are noted using a greek letter defined in ConoServer.
