# Supplementary material for: High-throughput identification of novel conotoxins from the Chinese tubular cone snail (Conus betulinus) by multi-transcriptome sequencing
Source: Gigascience. 2016 Apr 14;5:17. doi: 10.1186/s13742-016-0122-9 (PMC4832519; doi:10.1186/s13742-016-0122-9)
Supplement: Additional file 5: — Primer sequences used for RT-PCR (5’ to 3’). (DOCX 13 kb) [file 13742_2016_122_MOESM5_ESM.docx]

**Additional file 5: Primer sequences used for RT-PCR (5’ to 3’)**

| **Conopeptide** | **Primer sequences (forward and reverse)** |
| --- | --- |
| Bt018 | F-ATTCCTGCCTTTCAACCCTAACC |
|  | R-AAACCCTACGATTCTATGATGACTTTC |
| Bt044 | F-AGGCAGTCGCCAGGTAAATC |
|  | R-CTACTACAAGAATCCGTGAAGCAA |
| Bt054 | F-ATGGTGGCGACGGTTATT |
|  | R-GATCGGCGGCGTCTATT |
| Bt055 | F-CGTTTGATGTCACTCTGCTGCTT |
|  | R-CAACTTTATTGCCACTTTGCTTCC |
| Bt082 | F-CGATAGGGACCAGGACCTAGCAG |
|  | R-CGACCACAGGGTATTGGCAGAGT |
| β-Actin | F-TCACCCACACTGTGCCCATCTACGA |
|  | R-CAGCGGAACCGCTCATTGCCAATGG |
