# Supplementary figures and images for: High-throughput identification of novel conotoxins from the Chinese tubular cone snail (Conus betulinus) by multi-transcriptome sequencing
Source: Gigascience. 2016 Apr 14;5:17. doi: 10.1186/s13742-016-0122-9 (PMC4832519; doi:10.1186/s13742-016-0122-9)

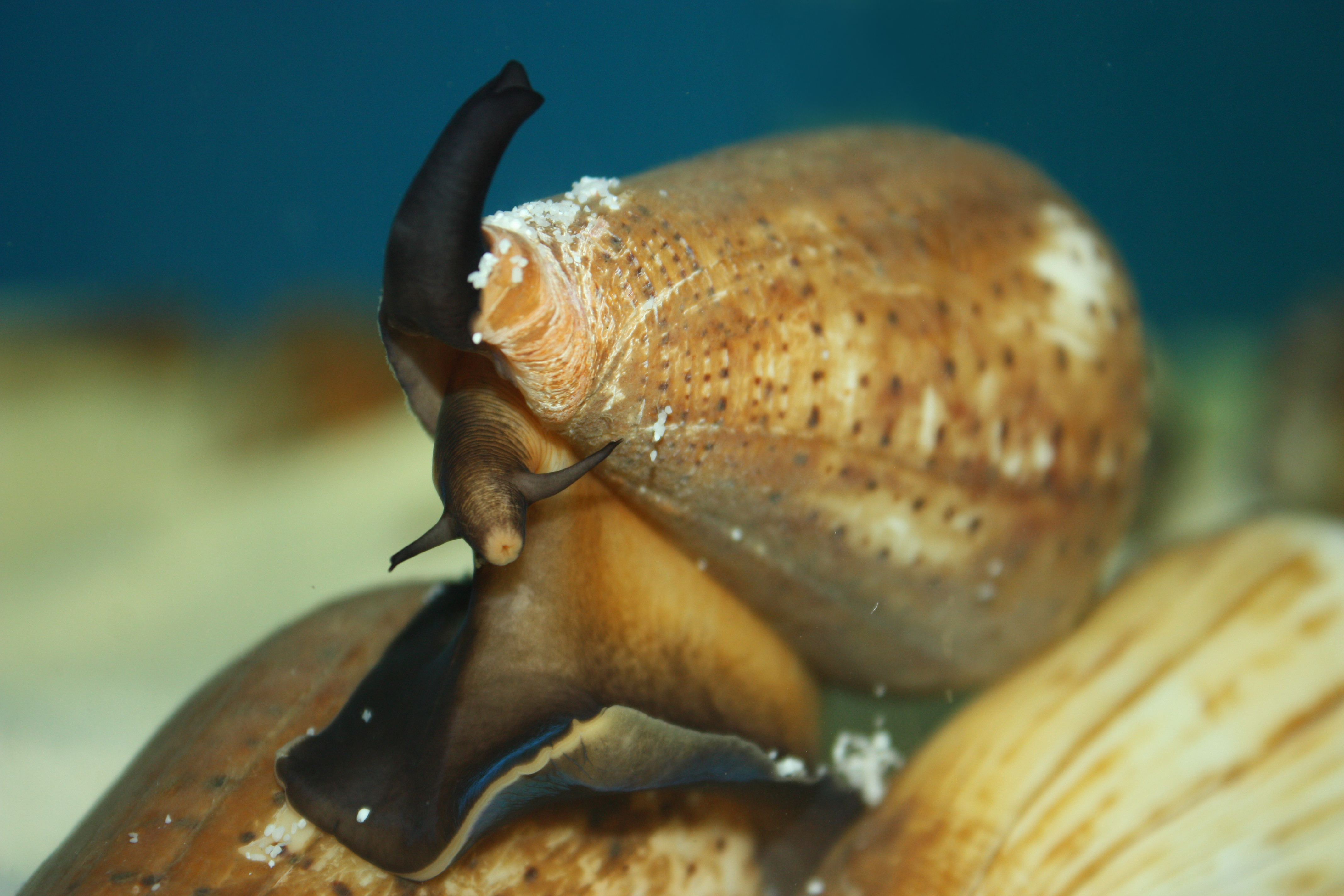

Supplement: Additional file 6: — A picture of Conus betulinus (Linnaeus) native to the South China Sea. (JPG 4261 kb) [file 13742_2016_122_MOESM6_ESM.jpg]
